# Left ventricular noncompaction—a rare cause of triad: heart failure, ventricular arrhythmias, and systemic embolic events: a case report 

**DOI:** 10.1186/s13256-021-02862-x

**Published:** 2021-06-08

**Authors:** Despina Toader, Alina Paraschiv, Petrișor Tudorașcu, Diana Tudorașcu, Constantin Bataiosu, Adrian Balșeanu

**Affiliations:** 1 Craiova Cardiology Center - EuroEchoLab, Craiova, Romania; 2 Craiova Cardiology Center - Cardiology Department, Craiova, Romania; 3 Faculty of Medicine - Internal Medicine, Department, The University of Medicine and Pharmacy Craiova, Craiova, Romania; 4 Craiova Cardiology Center - Electrophysiology Department, Craiova, Romania; 5 Faculty of Medicine- Physiology Department, The University of Medicine and Pharmacy Craiova, Craiova, Romania

**Keywords:** Left ventricular noncompaction, Dilated cardiomyopathy, Ventricular arrhythmias, Cardioverter implant, Case presentation

## Abstract

**Background:**

Left ventricular noncompaction is a rare cardiomyopathy characterized by a thin, compacted epicardial layer and a noncompacted endocardial layer, with trabeculations and recesses that communicate with the left ventricular cavity. In the advanced stage of the disease, the classical triad of heart failure, ventricular arrhythmia, and systemic embolization is common. Segments involved are the apex and mid inferior and lateral walls. The right ventricular apex may be affected as well.

**Case presentation:**

A 29-year-old Caucasian male was hospitalized with dyspnea and fatigue at minimal exertion during the last months before admission. He also described a history of edema of the legs and abdominal pain in the last weeks. Physical examination revealed dyspnea, pulmonary rales, cardiomegaly, hepatomegaly, and splenomegaly. Electrocardiography showed sinus rhythm with nonspecific repolarization changes. Twenty-four-hour Holter monitoring identified ventricular tachycardia episodes with right bundle branch block morphology. Transthoracic echocardiography at admission revealed dilated left ventricle with trabeculations located predominantly at the apex but also in the apical and mid portion of lateral and inferior wall; end-systolic ratio of noncompacted to compacted layers > 2; moderate mitral regurgitation; and reduced left ventricular ejection fraction. Between apical trabeculations, multiple thrombi were found. The right ventricle had normal morphology and function. Speckle-tracking echocardiography also revealed systolic left ventricle dysfunction and solid body rotation. Abdominal echocardiography showed hepatomegaly and splenomegaly. Abdominal computed tomography was suggestive for hepatic and renal infarctions. Laboratory tests revealed high levels of N-terminal pro-brain natriuretic peptide and liver enzymes. Cardiac magnetic resonance evaluation at 1 month after discharge confirmed the diagnosis. The patient received anticoagulants, antiarrhythmics, and heart failure treatment. After 2 months, before device implantation, he presented clinical improvement, and echocardiographic evaluation did not detect thrombi in the left ventricle. Coronary angiography was within normal range. A cardioverter defibrillator was implanted for prevention of sudden cardiac death.

**Conclusions:**

Left ventricular noncompaction is rare cardiomyopathy, but it should always be considered as a possible diagnosis in a patient hospitalized with heart failure, ventricular arrhythmias, and systemic embolic events. Echocardiography and cardiac magnetic resonance are essential imaging tools for diagnosis and follow-up.

**Supplementary Information:**

The online version contains supplementary material available at https://doi.org/10.1186/s13256-021-02862-x.

## Introduction

Left ventricular noncompaction (LVNC) is rare congenital cardiomyopathy characterized by a double-layered aspect of the myocardium: a compacted epicardial layer and a noncompacted endocardial layer, with trabeculations and recesses that communicate with the left ventricular (LV) cavity [1]. Segments involved are the apex and mid inferior and lateral walls. Right ventricular (RV) apex may be involved as well. In some cases, trabeculations are presented in both LVNC and dilated cardiomyopathy (DCM).

The echocardiographic appearance of a patient with LVNC in an advanced stage of the disease might be similar to any cause of DCM [2]. When LV function is severely decreased, heart failure, ventricular arrhythmia, and systemic embolization may also be common [2]. This makes differentiation difficult.

## Case presentation

A 29-year-old Caucasian male was hospitalized with dyspnea and fatigue at minimal exertion during the last months before admission. He also described a history of edema of the legs and abdominal pain in the previous weeks.

Physical examination revealed a patient with orthopnea, crepitations on the lower pulmonary lobes, upper abdominal pain due to congestive hepatomegaly, and symmetrical edema of both lower extremities. Blood pressure at admission was 100/60 mmHg. Signs of LV enlargement were present with the cardiac impulse displaced in intercostal space VII, lateral to the left midclavicular line. Heart rate was 90 beats/minute, the first and second heart sounds were normal, and S3 and S4 gallop were present. Auscultation identified a grade III/VI systolic murmur at the upper left sternal border. Jugular venous pressure was increased. Abdominal palpation identified congestive hepatomegaly, splenomegaly, and also positive abdominal jugular reflux.

**Fig. 1:**
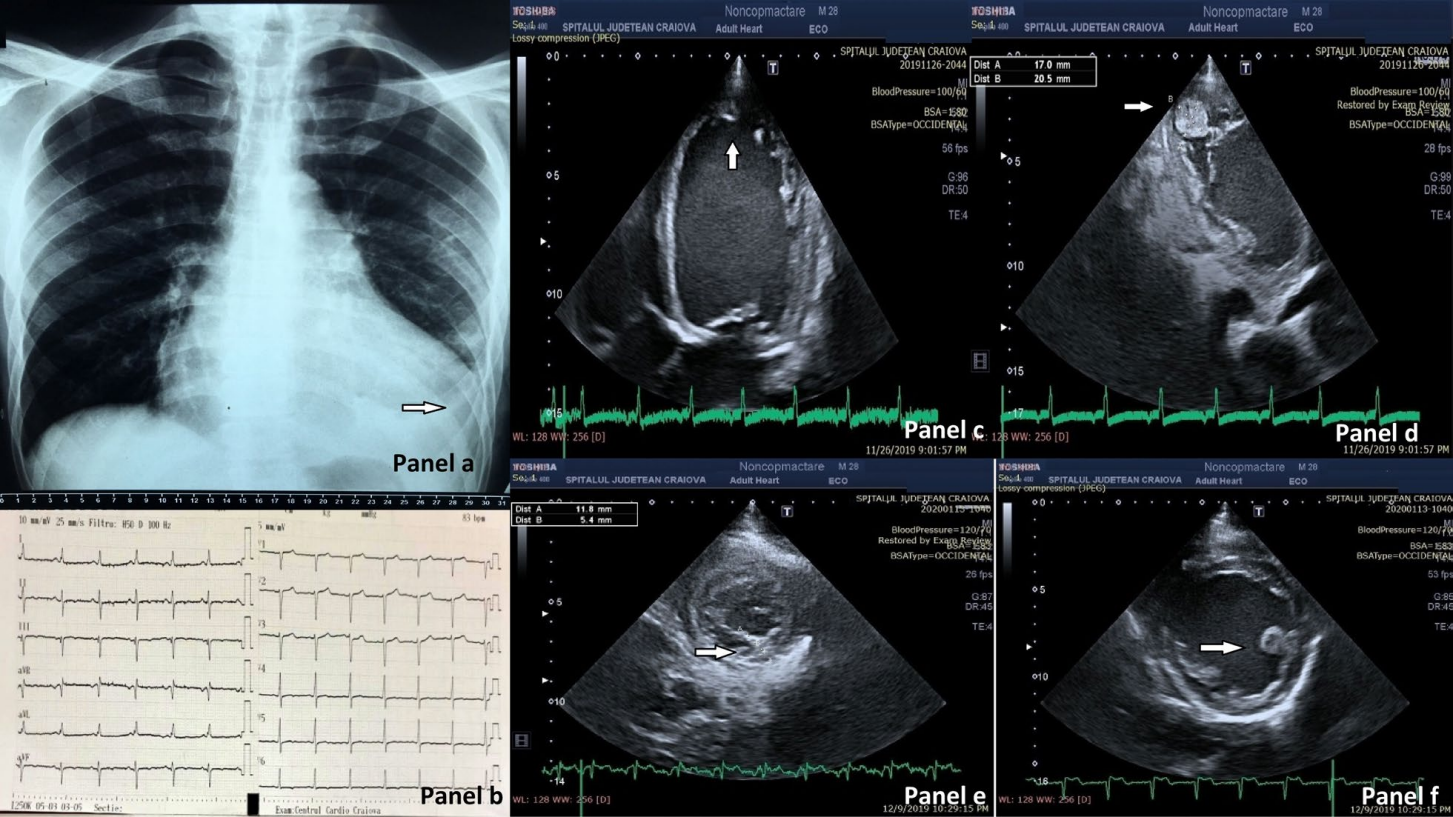
**a** Chest X-ray: cardiomegaly (arrow).** b** Twelve-lead electrocardiogram (ECG): sinus rhythm, normal QRS duration, and nonspecific repolarization abnormalities including T-wave flattening and inversion in the inferior and lateral leads II, III, aVF, V3–V6.** c** 2D Echocardiography: apical four-chamber (A4c) view showing dilated LV, trabeculation of apex, the apical segment of the interventricular septum, medium and apical segments of the lateral wall with two-layer aspect (arrow).** d** 2D Echocardiography: modified apical three-chamber (A3c) view with zoom showing trabeculation of the apex, medial, and apical segments of the inferolateral wall with thrombus between the recesses (arrow).** e** 2D Echocardiography: parasternal short-axis view at the intermediate level, between the papillary muscles and the apex revealing ratio between NC/C layers = 2.1 (arrow).** f** 2D Echocardiography: parasternal short-axis at the papillary muscles level showing dilated LV with posterior displacement of the papillary muscles (arrow)

Chest X-ray revealed cardiomegaly (Fig. 1a).

Twelve-lead electrocardiogram (ECG) showed sinus rhythm, normal QRS duration, and nonspecific repolarization abnormalities including T-wave flattening and inversion in the inferior and lateral leads II, III, aVF, V3–V6. (Fig. 1, Panel b).

Twenty-four-hour Holter monitoring identified ventricular tachycardia episodes with right bundle branch block (RBBB) morphology and frequent premature ventricular contractions (PVC).

Transthoracic echocardiographic (TTE) evaluation was obtained according to Recommendations for Cardiac Chamber Quantification by Echocardiography in Adults of the American Society of Echocardiography and the European Association of Cardiovascular Imaging 2015 [3].

**Fig. 2:**
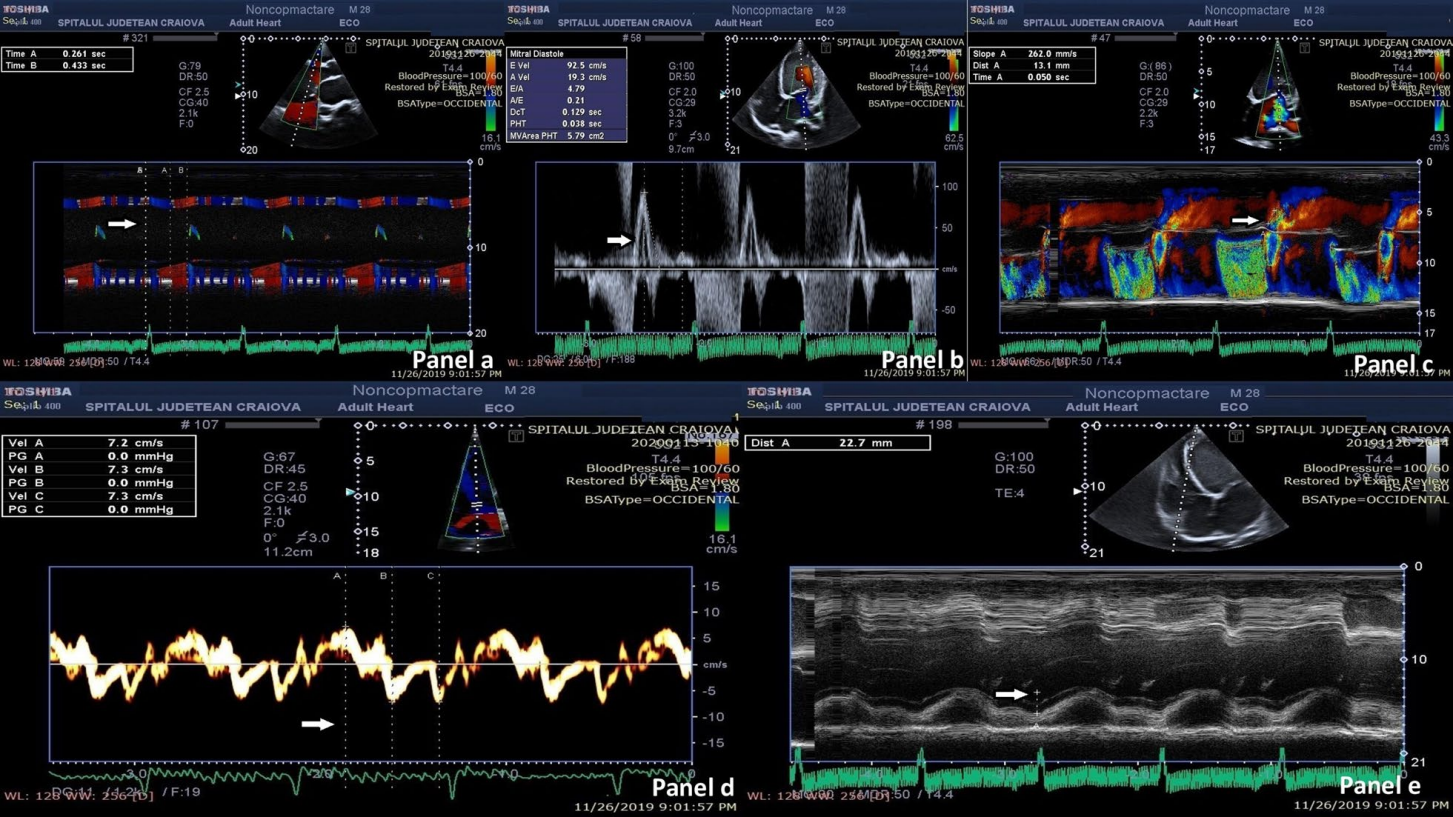
**a** Color M-mode; septal to posterior wall motion delay (SAPWD) calculation: 140ms. **b** Pulsed wave (PW) Doppler of mitral diastolic inflow, revealing a restrictive filling Pattern. **c** Color M-mode al the level of the mitral valve with velocity propagation (vp) measurement.** d** 2D tissue Doppler echocardiography with e wave velocity measurement.** e** M-mode echocardiography at the level of lateral tricuspid annulus providing tricuspid annular plane systolic excursion (TAPSE)

Two-dimensional (2D) TTE examination revealed a dilated and remodeled, spherical LV with morphological changes: a two-layered aspect of the myocardium with trabeculations located predominantly at the apex, but also in the apical and mid portion of the lateral and inferior wall (Fig. 1c, Additional files 1, 2). Multiple mobile thrombi were present between apical trabeculations (Fig. 1d, Additional file 3). The end-systolic ratio of noncompacted to compacted layer measured in the parasternal short-axis view was 2.1 (Fig. 1e). Color Doppler displayed flow within the deep intertrabecular recesses. Mitral annulus diameter dimension was 45 mm, the height of coaptation was 1.67 cm, and the tenting area was 6.72 cm^2^. Short-axis view examination showed papillary muscles posteriorly displaced (Fig. 1, Panel f). LV presented diffuse hypokinesia accentuated at the level of trabeculated zones, with asynergy of contraction, which was observed on 2D examination and in color M-mode; septal to posterior wall motion delay (SAPWD) was 140 ms (Fig. 2a). Simpson biplane method revealed an increased left atrium volume index (LAVi) of 44 ml/m^2^.

2D Color Doppler echocardiography and continuous Doppler (CW) echocardiography 2D Color Doppler echocardiography and continuous Doppler (CW) echocardiography revealed functional mitral regurgitation due to LV remodeling (Additional file 4).

For LV systolic function, many methods were used, trying to differentiate between the LVNC and a DCM with trabeculations in the LV. The biplane Simpson method revealed increased volumes: LV end-diastolic volume (LVDV) 296.6 ml, LV end-systolic volume (LVSV) 221 ml, decreased LVEF: 25%, and cardiac index (CI) 3.06 ml/minute/m^2^.

Tissue Doppler echocardiography also showed decreased velocities at the level of septal (7 cm/second) (Fig. 2d) and lateral (5 cm/second) mitral annulus.

LV diastolic function was evaluated using pulsed wave (PW) Doppler of mitral diastolic inflow, revealing a restrictive filling pattern (Fig. 2b), color M-mode an E/vp ratio of 3.5 (Fig. 2c), and tissue Doppler echocardiography an E/e ratio of 15.41 (Fig. 2d). These measurements revealed an increased LV filling pressure.

**Fig. 3:**
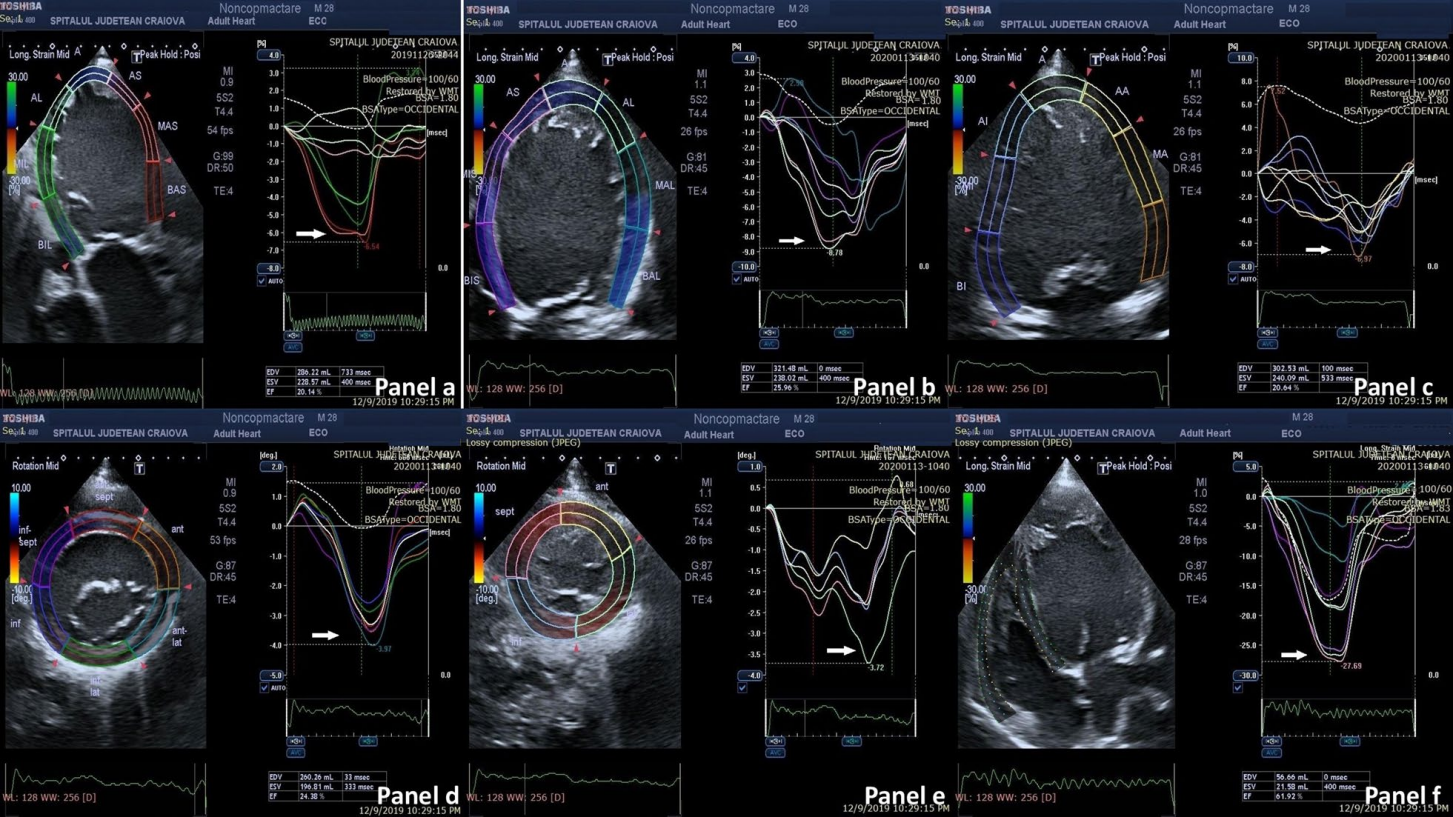
**a** 2D Speckle Tracking Echocardiography (STE) A3c view, sowing decreased left ventricle (LV) global longitudinal strain (GLS) and left ventricle ejection fraction (LVEF).** b** 2D STE A4c view: decreased LV GLS and LVEF.** c** 2D STE A2c view: decreased LV GLS and LVEF.** d** 2D STE: peak radial strain at the LV basal level.** e** 2D STE: peak radial strain at the LV apex.** f** 2D STE: Right ventricle (RV) GLS

On speckle-tracking echocardiography (STE) examination, global longitudinal strain (GLS) was decreased (7.43%), as well as LVEF (22%) (Fig. 3a–c). Twist motion was calculated using the difference in peak rotation between the basal and apical short-axis planes of LV: instantaneous peak LV twist (apical LV peak rotation – basal LV peak rotation). A decreased LV twist of 0.24 was found (Fig. 3d, e). The RV had normal morphology, dimension, and function; fractional area change (FAC), tricuspid annular plane systolic excursion (TAPSE) (Fig. 2e), tissue Doppler velocities at the level of tricuspid annulus, GLS, and RVEF by STE (Fig. 3f) evaluations were within a normal range.

Tricuspid regurgitation was moderate, and inferior vena cava dilated, without inspiratory collapse. The estimated pulmonary artery systolic pressure (PASP) was 42 mmHg. Laboratory tests detected high levels of N-terminal pro-brain natriuretic peptide (NT-pBNP) and liver enzymes.

Abdominal ultrasound examination showed hepatomegaly and splenomegaly. Abdominal computed tomography (CT) scan was suggestive of hepatic and renal infarctions. Angiography found no coronary lesions.

The patient was scheduled for cardiac magnetic resonance (CMR), which was possible after 1 month. This approach was taken into consideration for LVNC diagnosis certification and to differentiate it from DCM. During this time, the treatment included anticoagulant, antiarrhythmic, diuretic, beta blocker, and angiotensin-converting enzyme inhibitors.

**Fig. 4:**
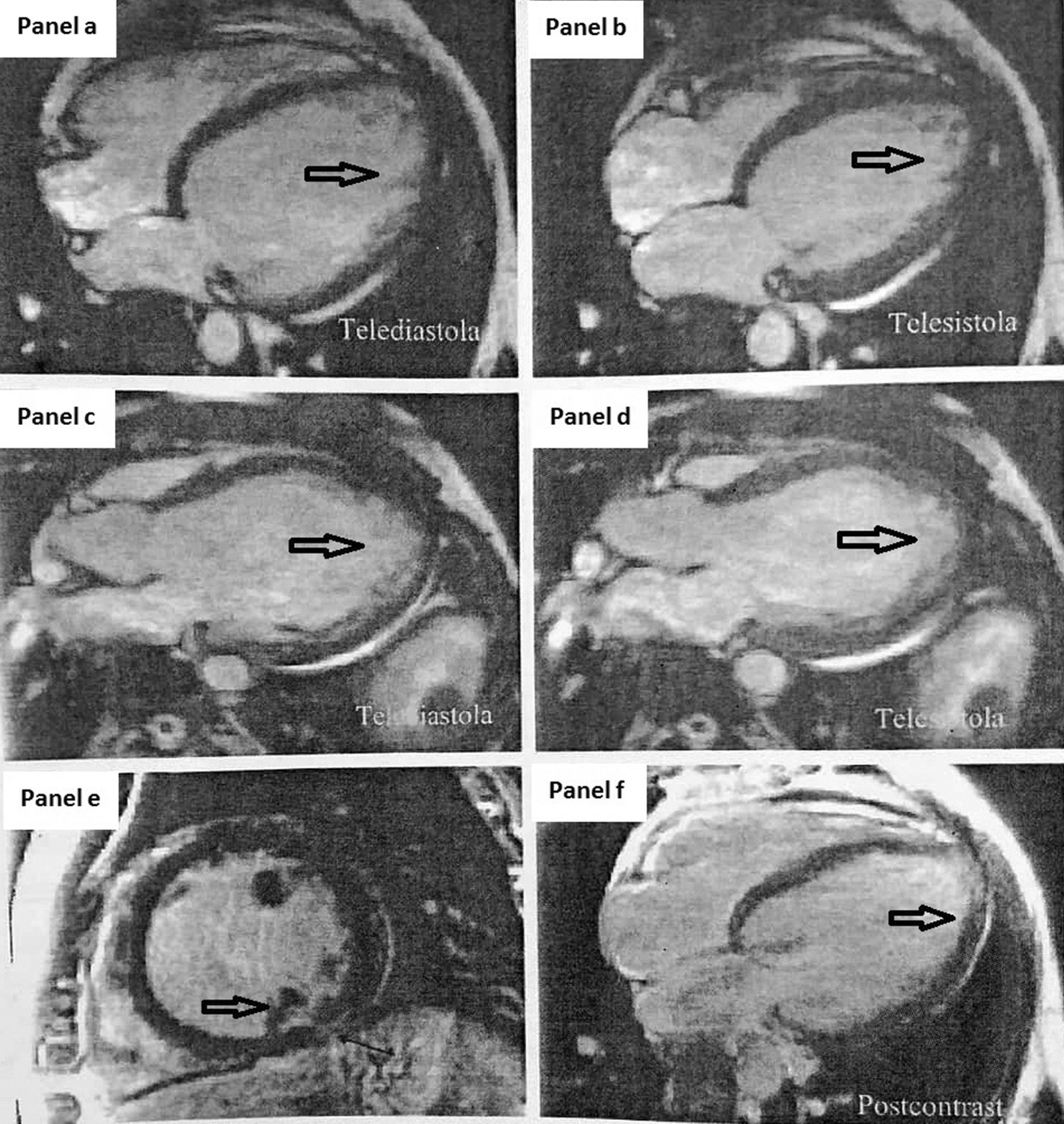
**a**–**d** Cardiac magnetic resonance (CMR) showing dilated LV with trabeculations, recesses, and two-layer aspect of the myocardium (arrows).** e** CMR: fragmentation aspect of papillary muscles (arrow).** f** CMR: Late contrast administration identifying a zone with suspicion of scar (fibrosis) or Fabry disease at the level of the subendocardial inferior and inferolateral wall (arrow)

The LVNC diagnosis was confirmed by CMR, that is, by the presence of trabeculations located from the medial to apical zones of LV (Fig. 4), two-layer aspect (Fig. 4a–d), and fragmentation aspect of papillary muscles (Fig. 4e). The maximal thickness of the myocardium was 8 mm, at the basal anteroseptal level. Examination detected no edema areas, and the following values: LVDV 375 ml (197 ml/m^2^), LVSV 245 ml (129 ml/m^2^), LVEF 35%, LVMi 230 g (121 g/m^2^), RVDV 152 ml (80 ml/m^2^), RVSV 44 ml (23 ml/m^2^), and RVEF 71% (Fig 4a, b, f). The ratio of noncompacted myocardium to compacted myocardium was greater than 2.3 during diastole, and trabeculated left ventricular mass accounted for more than 20% of the total mass. The RV had standard dimensions and systolic and diastolic function, without kinetics modifications. The right ventricle ejection fraction (RVFE) was 71%. Mitral regurgitation and tricuspid regurgitation were moderate. Late contrast administration identified a zone with suspicion of scar (fibrosis) or Fabry disease at the level of subendocardial inferior and inferolateral wall (Fig. 4d–f). Genetic tests were negative for Fabry disease, as well as for Gaucher and Niemann–Peck diseases.

Familial screening of first-degree relatives was also negative for LVNC, DCM, and congenital heart disease.

The second evaluation of the patient was performed after 2 months of anticoagulation and treatment of heart failure. He had no signs of heart failure. Echocardiographic examination detected no thrombi in the apex of the left ventricle between trabeculations (Additional files 5, 6). A cardioverter defibrillator was implanted for prevention of sudden cardiac death (Additional file 7).

The patient was proposed for cardiac transplantation.

## Discussions

Left ventricular noncompaction (LVNC) is a rare cardiomyopathy. Prevalence is less than 0.02% [1] and is male predominant [1]. It can occur in isolation or association with other pathologies of the heart, with or without associated gene mutations [1, 2]. LVNC may be familial with an autosomal dominant mode of inheritance or sporadic [1, 2]. The sporadic occurrence was found in up to 60–70% of the cases [1]. Prominent trabeculations on the endocardial surface with deep recesses extending into the left ventricle (LV) wall characterize the disease [1].

Genetic studies showed that mutations were significantly more frequent in sarcomere genes (82%) [2]. Non-sarcomere gene mutations might be identified in a minority of genetic cases and rare cases presenting with complex genotypes [2]. Some genes involved in LVNC are also involved in hypertrophic cardiomyopathy and dilated cardiomyopathy (genes that encode sarcomere proteins) [2]. LV systolic dysfunction seems to be more frequent in genetic than in sporadic cases. In a patient with a myocardial phenotype of LVNC, the association between a genetic mutation and LV dysfunction as a risk factor of the worse outcome supports the hypothesis that LVNC is a genetically determined cardiomyopathy [2].

In 2006, The American Heart Association (AHA)-led working groups and councils classified LVNC as congenital genetic cardiomyopathy [4]. In 2008, the European Society of Cardiology Working Group on Myocardial and Pericardial Diseases considered it an unclassified familial cardiomyopathy [5].

In embryogenesis, before the development of coronary arteries, the myocardium has a trabeculated aspect. The process of trabecular compaction in the human heart starts at the base of the LV and progresses toward the apex [6]. LVNC is considered to be the consequence of early cessation of compaction during embryogenesis. This anomaly may be why ventricular noncompaction usually involves the apical regions [6]. The result is an epicardial layer that is compacted and an endocardial layer with prominent trabeculae and deep intertrabecular recesses that communicate with the left ventricle cavity [6]. Segments involved in more than 80% of patients are apical and mid-ventricular inferior wall segments and the mid-ventricular lateral wall [6].

In LVNC, there is a mismatch between the myocardial mass and the number of capillaries, leading to hypoperfusion of the endocardial myocardium despite normal epicardial coronary arteries [6]. The disease is usually associated with reduced ejection fraction and systolic dysfunction, attributed to hypoperfusion and to asynchronism of contraction between the compacted and noncompacted myocardial layers. Hypokinesia was observed both in compacted and in noncompacted segments [6]. Papillary muscles are not well developed. The right ventricular apex may be involved as well [6]. Trabeculations diminish ventricular compliance, leading to diastolic dysfunction, that is, an abnormal relaxation or a restrictive filling pattern [6]. Ischemia is responsible for the progressive fibrosis, which also contributes to the decrease of LVEF and predisposes to ventricular arrhythmias [6].

Patients with LVNC can be asymptomatic or may present symptoms of heart failure (HF), supraventricular and ventricular arrhythmias, thromboembolic events, and sudden cardiac death. Symptoms of HF occur in more than half of the patients with LVNC, LV dysfunction being reported in up to 84% of them. In total, 36% of study patients had heart failure of function class III and class IV [6]. Systemic embolic events are frequent in patients with LVNC. The incidence of thromboembolic complications ranges from 5% to 38% [6]. Cardioembolic events are secondary to mural thrombi formed in the recesses between trabeculations in the noncompacted myocardium, but also to the depressed LVEF or the development of atrial fibrillation [6]. Systemic embolic complications secondary to LVNC are cerebral, myocardial, renal, and mesenteric [6].

ECG can be normal in 13% of cases [6]. Early repolarization abnormalities were found in 40% of patients with LVNC, and QTc prolongation in over 50% of patients [6]. Repolarization disturbances predispose patients to malignant ventricular tachyarrhythmias and sudden cardiac death [6]. Atrial fibrillation has been reported in over 25% of cases, and ventricular tachyarrhythmias in 47% [6]. Paroxysmal supraventricular tachycardia and complete heart block have also been reported [6].

According to the 2006 AHA scientific statement, the LVNC cardiomyopathy diagnosis is obtained by using imaging techniques, that is, TTE, CMR imaging, or LV angiography with ventriculography, but no specific guidelines or imaging criteria recommendations are formally provided [4].

The most used method of diagnosis is echocardiography [7]. There are two sets of echocardiographic criteria: the Jenni criteria focused on the presence of a two-layered structure [8], and the Chin criteria focused on the depth of the recess compared with the height of the trabeculations [9]. Jenni criteria [8] are the most accepted validated echo criteria and consist of evidence of a two-layer structure: a compacted thin epicardial layer and a thicker noncompacted endocardial layer with prominent trabeculation and deep intertrabecular recesses. In the short-axis view, the end-systolic ratio of noncompacted to compacted layers > 2.0 is diagnostic [8]. Additional criteria that must be met include the absence of any coexisting cardiac abnormalities and color Doppler evidence of deep perfused intertrabecular recesses [8]. Chin criteria [9] considered for diagnosis are the presence of numerous, excessively prominent trabeculations and deep intertrabecular recesses with the ratio of the distance from the epicardial surface to the trough of the trabecular recesses and distance from the epicardial surface to the peak of trabeculation ≤ 0.5, assessed at end-diastole on short-axis parasternal views and/or apical views. It is also important that no other cardiac structural abnormalities be present [9]. Stollberger *et al.* defined LVNC as trabeculations >/ 3, prominent formations along the left ventricular endocardial border, located apically to the papillary muscles, visible in end-diastole, in one imaging plane, moving synchronously with the compacted myocardium, distinct from the papillary muscles, false tendons, or aberrant bands [10]. Ghebhard *et al.* considered compacted myocardium systolic thickness < 8 mm for diagnosis of LVNC [11].

In difficult cases, other echocardiographic techniques can be used for diagnosis: contrast enhancement, three-dimensional echocardiography, speckle tracking, and tissue Doppler imaging. Speckle-tracking echocardiography (STE) was used in borderline cases because LVNC affects the left ventricle twist [12]. In a normal heart, left ventricular twisting motion is caused by rotation in a clockwise direction (as seen from the apex) at the level of the mitral valve (basal level) and counterclockwise rotation of the apex (apical level). This movement contributes 60% to ejection fraction and 15% to fiber shortening. Left ventricular untwisting is involved in active diastolic filling [12]. In patients with LVNC, an abnormal rotation pattern was described, that is, with basal and apical rotation in the same direction, resulting in almost total absence of left ventricular twist. This rotation pattern was described by Dalen *et al.* in 2008 as left ventricular solid body rotation, and it was proposed as a sensitive and specific marker for LVNC diagnosis that could differentiate it from DCM [13]. It has also demonstrated its importance in prognosis: patients with rigid body rotation and noncompaction cardiomyopathy had a lower NYHA functional status as compared with patients without rigid body rotation [13]. Peters *et al.* found rigid body rotation in 53% of patients with LVNC, and they highlighted the importance of left ventricular twist evaluation in cardiomyopathies [14].

Echocardiography is the current gold standard for the diagnosis of this entity [14]. There are frequent doubtful cases that need multimodal confirmation (echocardiography and magnetic resonance imaging) [15].

CMR imaging is superior to echocardiography for the identification of noncompacted myocardium, with better image quality and increased sensitivity for identifying trabeculations, particularly at end-diastole [15]. Petersen *et al.* criteria elaborated in 2005 are accepted cardiac MRI diagnostic parameters for the evaluation of LVNC, that is, the presence of two distinct myocardial layers and marked trabeculations with deep intertrabecular recesses within the inner noncompacted layer; a noncompacted/compacted myocardium ratio > 2.3 at end-diastole was considered suggestive [15]. Additional parameters have been introduced for the assessment of LVNC. Jacquier *et al.* considered a trabeculated left ventricular mass > 20% of the global left ventricular mass measured at end-diastole as a sensitive and specific finding for the diagnosis of LVNC [16]. Grothoff *et al.* introduced a quantitative measurement for LVNC diagnosis: trabeculated mass should represent > 25% of the LV global mass and > 15 g/m^2^ [17]. It was suggested that CMR should play a significant role in the evaluation when the diagnosis by the echocardiogram is not confirmed, a good-quality echocardiogram cannot be obtained, and/or the degree of fibrosis may help in delineating the severity of the disease [17].

Genes coding for sarcomere proteins, ion channels, and cellular signaling pathways implicated in other cardiomyopathies have been associated with LVNC [18]. LVNC may appear in isolation or can be associated with other cardiomyopathies, including DCM, hypertrophic cardiomyopathy (HCM), restrictive cardiomyopathy, arrhythmogenic right ventricular cardiomyopathy, or congenital heart disease, such as Ebstein anomaly [18]. It might also be associated with Barth syndrome, mitochondrial disorders, and myotonic dystrophy [18]. Mutations in genes MYH7, MYBPC3, and TTN are the most common in patients with LVNC [18].

The 2011 HRS/EHRA Expert Consensus Statement on the State of Genetic Testing for the Channelopathies and Cardiomyopathies states that, owing to the low rate of a positive genetic test in index cases, the utility of genetic testing for the definitive diagnosis and care of the index case is of limited use [19].

Family screening in patients diagnosed with LVNC can help determine if a cardiac abnormality is sporadic or familial. Relatives may present with isolated LVNC and with other forms of congenital heart disease or cardiomyopathy with or without LVNC [19]. For family members in whom trabeculations or LVNC are identified, close clinical surveillance should be recommended [19].

There is no therapy specific for patients with LVNC [20]. Data from randomized controlled trials to guide the management of LVNC cardiomyopathy are limited, and interventions are focused on complications, that is, heart failure, systemic embolism, and sudden cardiac death [20]. The same treatment is recommended to patients with DCM and reduced ejection fraction [20]. Prevention of systemic embolism is an important management goal in these patients [20]. Whether anticoagulants should be administered to every LVNC patient is, however, still debated [20]. Anticoagulation therapy must be targeted to the individual patient after careful assessment of the benefit and risks. Oral anticoagulation therapy (target INR 2.0–3.0) was recommended in patients with impaired systolic function (LV ejection fraction ≤ 40%), previous history of embolism, transient ischemic attack, atrial fibrillation, and intracardiac thrombi identified on echocardiogram or another cardiac imaging modality [20]. Otherwise, risk assessment based on CHADS2/CHADS2-Vasc scores as guidance and preference of the patient is recommended [20].

Patients with LVNC and sustained ventricular tachycardia or ventricular fibrillation require cardioverter-defibrillator (ICD) implantation. These patients are at higher risk for SCD, even with normal EF [21]. This should be based on current ICD primary and secondary prevention guidelines [21]. ICD for primary prevention of sudden cardiac death is indicated for patients with LVNC who present with cardiomyopathy and ejection fraction ≤ 35% [21]. Patients with malignant ventricular tachyarrhythmia should undergo ICD implantation for secondary prevention [21].

Prognosis is proportional to the severity of systolic dysfunction of the left ventricle [22]. In 2012, Greutmann *et al.* found that NYHA class of heart failure >/ 3 and cardiovascular complications at presentation were strong predictors for adverse outcomes [22]. In 2020, a systematic review and meta-analysis of observational studies of Aung N. *et al.* found that, compared with DCM, patients with LVNC have similar risks of cardiovascular mortality, all-cause mortality, thromboembolic complications, and ventricular arrhythmia. The most important predictor of worse outcomes in patients with LVNC was low LVEF [23].

## Conclusion

We presented a rare case of heart failure in a patient with left ventricular noncompaction complicated with heart failure, ventricular tachycardia, and systemic embolization. LVNC is a rare cardiomyopathy and should always be considered as a possible diagnosis in a patient with the echocardiographic feature of dilatation and trabeculation of left ventricle.

The differentiation between LVNC and DCM in some patients remains challenging. A gold standard for diagnostic criteria has not been established. Echocardiogram is the initial study of choice, and if the diagnosis is indeterminate, a CMR may be the reasonable next test, which will also permit fibrosis assessment.

This case emphasizes the importance of imaging techniques, that is, echocardiography and CMR in early diagnosis, management, and follow-up.

## Supplementary Information

**Additional file 1.** Echocardiography: A4c view: trabeculation of the apex, medial, and apical segments of the anterolateral wall with two-layer aspect, hypokinesia, spontaneous contrast, and mobile thrombus at the level of the apex.

**Additional file 2.** Echocardiography A3c view: trabeculation of the apex, medial, and apical segments of the inferolateral wall with hypokinesia.

**Additional file 3.** Echocardiography: parasternal short-axis view at the level of the apex: trabeculation, spontaneous contrast, and multiple mobile thrombi.

**Additional file 4.** Echocardiography: A4c view, color Doppler: functional mitral regurgitation trabeculation of the apex, medial, and apical segments of the inferolateral wall with two-layer aspect, hypokinesia.

**Additional file 5.** Echocardiographic evaluation after 2 months of anticoagulation therapy, A4c view: trabeculation of the apex, medial, and apical segments of the inferolateral wall with two-layer aspect, hypokinesia, without thrombus at the level of the apex.

**Additional file 6.** Echocardiographic evaluation after 2 months of anticoagulation therapy: parasternal short axis-view at the level of the LV apex: trabeculation without thrombus.

**Additional file 7.** Echocardiographic A4c view with focalization on the right ventricle (RV): device, normal morphology, and function of the RV, cardioverter defibrillator.

## Data Availability

The authors declare that data supporting the findings of this study are available within the article and its Additional files.

## Abbreviations

ALT: Alanine transaminase
AST: Aspartate transaminase
CI: Cardiac index
CMR: Cardiac magnetic resonance
CT: Computed tomography
CW Doppler: Continuous wave Doppler
DCM: Dilated cardiomyopathy
ECG: Electrocardiography
FAC: Fractional area change
GLS: Global longitudinal strain
HCM: Hypertrophic cardiomyopathy
ICD: Implanted cardioverter defibrillator
LAVi: Left atrial volume index
LV: Left ventricle
LVEF: Left ventricle ejection fraction
LVDV: Left ventricle end-diastolic volume
LVSV: Left ventricle end-systolic volume
LVNC: Left ventricular noncompaction
LVMi: Left ventricle mass index
NTpBNP: N-terminal pro-brain natriuretic peptide
PASP: Pulmonary artery systolic pressure
PE: Physical examination
PVC: Premature ventricular contractions
PW Doppler: Pulsed-wave Doppler
RBBB: Right bundle branch block
RV: Right ventricle
RVEF: Right ventricle ejection fraction
RVDV: Right ventricle end-diastolic volume
RVSV: Right ventricle end-systolic volume
SAPWD: Septal to posterior wall motion delay
STE: Speckle-tracking echocardiography
TAPSE: Tricuspid annular plane systolic excursion
TTE: Transthoracic echocardiography

## References

[1] Wengrofsky P, Armenia C, Oleszak F,* et al*. Left ventricular trabeculation and noncompaction cardiomyopathy: a review. EC Clin Exp Anat. 2019;2(6):267–83.

[2] Oechslin E, Jenni R. Left ventricular noncompaction from physiologic remodeling to noncompaction cardiomyopathy. J Am Coll Cardiol. 2018. 10.1016/j.jacc.2017.12.031. 29447732

[3] Lang MR, Badano LP, Mor-Avi V,* et al*. Recommendations for cardiac chamber quantification by echocardiography in adults: an update from the American Society of Echocardiography and the European Association of cardiovascular imaging. J Am Soc Echocardiogr. 2015. 10.1016/j.echo.2014.10.003. 25559473

[4] Maron BJ, Towbin JA, Thiene G,* et al*. Contemporary definitions and classification of the cardiomyopathies: an American Heart Association Scientific Statement from the Council on Clinical Cardiology, Heart Failure and Transplantation Committee; Quality of Care and Outcomes Research and Functional Genomics and Translational Biology Interdisciplinary Working Groups; and Council on Epidemiology and Prevention. Circulation. 2006;113:1807–16. 10.1161/CIRCULATIONAHA.106.174287. 16567565

[5] Elliott P, Andersson B, Arbustini E,* et al*. Classification of the cardiomyopathies: a position statement from the European Society Of Cardiology Working Group on Myocardial and Pericardial Diseases. Eur Heart J. 2008;29(2):270–6. 10.1093/eurheartj/ehm342. 17916581

[6] Weiford BC, Subbarao VD, Mulhern KM. Noncompaction of the ventricular myocardium. Circulation. 2004;109:2965–71. 10.1161/01.CIR.0000132478.60674. 15210614

[7] Thomas DE, Wheeler R, Yousef ZR, Masani ND. The role of echocardiography in guiding management in dilated cardiomyopathy. Eur J Echocardiogr. 2009;10:iiil15-iii21. 10.1093/ejechocard/jep158. 19889654

[8] Jenni R, Oechslin E, Schneider J,* et al*. Echocardiographic and pathoanatomical characteristics of isolated left ventricular non-compaction: a step towards classification as a distinct cardiomyopathy. Heart. 2001;86:666–71. 10.1136/heart.86.6.666. PMC1730012 11711464

[9] Chin TK, Perloff JK, Williams RG,* et al*. Isolated noncompaction of left ventricular myocardium. A study of eight cases. Circulation. 1990;82(2):507–13. 10.1161/01.cir.82.2.507. 2372897

[10] Stollberger C, Finsterer J. Left ventricular hypertrabeculation/noncompaction. J Am Soc Echocardiogr. 2004;17:91–100. 10.1016/S0894-7317(03)00514-5. 14712196

[11] Gebhard C, Stahli BE, Greutmann M,* et al*. Reduced left ventricular compacta thickness: a novel echocardiographic criterion for non-compaction cardiomyopathy. J Am Soc Echocardiogr. 2012;25(10):1050–7. 10.1016/j.echo.2012.07.003. 22883316

[12] Niemann M, Liu D, Hu K,* et al*. Echocardiographic quantification of regional deformation helps to distinguish isolated left ventricular non-compaction from dilated cardiomyopathy. Eur J Heart Fail. 2012;14:155–61. 10.1093/eurjhf/hfr164. 22219500

[13] van Dalen BM, Caliskan K, Soliman OII,* et al*. Left ventricular solid body rotation in non- compaction cardiomyopathy: a potential new objective and quantitative functional diagnostic criterion? Eur J Heart Fail. 2008;10:1088–93. 10.1016/j.ejheart.2008.08.006. 18815069

[14] Peters F, Khandheria BK, Libhaber E,* et al*. Left ventricular twist in left ventricular noncompaction. Eur Heart J Cardiovasc Imaging. 2014;15:48–55. 10.1093/ehjci/jet076. 23793875

[15] Petersen SE, Selvanayagam JB, Wiesmann F,* et al*. Left ventricular non-compaction: insights from cardiovascular magnetic resonance imaging. J Am Coll Cardiol. 2005;46:101–5. 10.1016/j.jacc.2005.03.045. 15992642

[16] Jacquier A, Thuny F, Jop B,* et al*. Measurement of trabeculated left ventricular mass using cardiac magnetic resonance imaging in the diagnosis of left ventricular non-compaction. Eur Heart J. 2010;31(9):1098–104. 10.1093/eurheartj/ehp595. 20089517

[17] Grothoff M, Pachowsky M, Hoffmann J,* et al*. Value of cardiovascular MR in diagnosing left ventricular non-compaction cardiomyopathy and in discriminating between other cardiomyopathies. Eur Radiol. 2012;22(10):2699–709. 10.1007/s00330-012-2554-7. PMC3486997 22772366

[18] van Waning JI, Caliskan K, Hoedemaekers YM,* et al*. Genetics, clinical features and long-term outcome of noncompaction cardiomyopathy. J Am Coll Cardiol. 2018;71:711–22. 10.1016/j.jacc.2017.12.019. 29447731

[19] Ackerman MJ, Priori SG, Willems S,* et al*. HRS/EHRA Expert Consensus Statement on the State of Genetic Testing for the Channelopathies and Cardiomyopathies This document was developed as a partnership between the Heart Rhythm Society (HRS) and the European Heart Rhythm Association (EHRA). Europace. 2011;13:1077–109. 10.1093/europace/eur245. 21810866

[20] Bennett CE, Freudenberger R. The current approach to diagnosis and management of left ventricular noncompaction cardiomyopathy: review of the literature. Cardiol Res Pract. 2016;2016:7. 10.1155/2016/5172308. PMC4737020 26881173

[21] Silvia G, Priori SG, Blomstro C, Andrea Mazzanti A,* et al*. ESC Guidelines for the management of patients with ventricular arrhythmias and the prevention of sudden cardiac death The Task Force for the Management of Patients with Ventricular Arrhythmias and the Prevention of Sudden Cardiac Death of the European Society of Cardiology (ESC) Endorsed by: Association for European Paediatric and Congenital Cardiology (AEPC). Eur Heart J. 2015;36:2793–867. 10.1093/eurheartj/ehv316. 26320108

[22] Greutmann M, Mah ML, Silversides CK,* et al*. Predictors of adverse outcome in adolescents and adults with isolated left ventricular noncompaction. Am J Cardiol. 2012;109:276–81. 10.1016/j.amjcard.2011.08.043. 22036106

[23] Aung N, Doimo S, Ricci F,* et al*. Prognostic significance of left ventricular noncompaction systematic review and meta-analysis of observational studies. Circ Cardiovasc Imaging. 2020;13:e009712. 10.1161/CIRCIMAGING.119.009712. PMC7012350 31959004

